# Guiding practice principles for clinicians who work with Indigenous people

**DOI:** 10.3389/fped.2025.1526753

**Published:** 2025-06-17

**Authors:** André Schultz, Anne B. Chang, Donna M. Mason, Julie M. Marchant, Lesley A. Versteegh, Maree Toombs, The CRE Extended Group, JM Atack

**Affiliations:** ^1^ Wal-yan Respiratory Research Centre, The Kids Research Institute, Perth, WA, Australia; ^2^ Division of Paediatrics, Faculty of Medicine, University of Western Australia, Perth, WA, Australia; ^3^ Department of Respiratory and Sleep Medicine, Perth Children’s Hospital, Perth, WA, Australia; ^4^ Australian Centre for Health Services Innovation, Queensland University of Technology, Brisbane, QLD, Australia; ^5^ NHMRC Centre for Research Excellence in Paediatric Bronchiectasis (AusBREATHE), Child Health Division, Menzies School of Health Research, Charles Darwin University, Darwin, NT, Australia; ^6^ Indigenous Respiratory Outreach Care (IROC), Children’s Health Queensland, Brisbane, QLD, Australia; ^7^ School of Public Health, University of Sydney, Sydney, NSW, Australia

**Keywords:** Indigenous, First Nations, children, cultural safety, respiratory, lung health

## Abstract

Culturally safe healthcare approaches are important to improve outcomes of Indigenous people. Non-Indigenous clinicians are often ill-prepared to provide such healthcare. The NHMRC Centre for Research Excellence (CRE) especially for First Nations Children has been studying for several years how to improve clinical care for Indigenous children with respiratory disease in hospital, clinic, urban, rural and remote settings. At a CRE meeting in 2023 key themes were identified based on what we have learned. Themes were informed by research conducted by the CRE and supplemented by relevant manuscripts known to CRE members. This manuscript provides practical information to aid clinicians in providing culturally safe healthcare to Indigenous people. In brief, the provision of health information that is relevant and understandable to Indigenous patients and their families is critical for ensuring condition-specific health literacy and to allow Indigenous patients to gain autonomy over medical care provided to them and their children. Methods to facilitate effective communication between healthcare providers and patients, and the creation of a culturally safe healthcare environments are discussed. The manuscript will be of practical use to clinicians and translatable to other areas of health care.

## Introduction

Health outcomes for Indigenous people are substantially worse than encountered in the general Australian population. Life expectancy is 9.5–10.6 years lower and the disease burden from conditions that cause illness and disability is 2.3 times higher in Indigenous than non-Indigenous Australians (1). The differences are also stark in the field of respiratory medicine. Acute and chronic respiratory diseases are almost 10-times more common in Indigenous than non-Indigenous children, more than 25% of Indigenous adults report having a chronic respiratory disorder, and adults with bronchiectasis die approximately 22-years earlier than other Australians with this diagnosis (2–4). A national “closing the gap” strategy is addressing this inequality in health outcomes in Australia (5). Whilst a high-level national strategy is essential, individual non-Indigenous clinicians still need to be well equipped to provide care for Indigenous patients. Not providing effective and the best possible clinical care whilst waiting for generic improvement in social determinants adds further to the disadvantage faced by Indigenous people (3, 6). When clinical care is of high quality, evidence-based, effective and culturally- safe, significant improvement in lung function has been documented (3, 7). This may be as (or more) effective as city-based tertiary care and is likely to be preferred by families (3, 7). A culturally-safe patient experience in clinical care can be achieved by: (i) demonstrating respect for an individual's (or family or community) identity and culture; (ii) an effective sharing of knowledge and experience; (iii) acknowledging any power imbalance and lack of social justice; and (iv) promoting agency and supporting individuals or families to achieve the healthcare services and the health outcomes they desire (8).

Our Australian National Health and Medical Research Council (NHMRC) Centre for Research Excellence (CRE) in Lung Health, especially for Indigenous children (9) has been studying how to improve clinical care for Indigenous patients for several years. These efforts have included purposely interviewing over 200 Indigenous parents and family members in hospital, clinic, urban, rural and remote settings to gain wisdom from them on how to become more effective clinicians (10–14). Many of our findings confirm knowledge generated previously in other disciplines, whilst new findings related to our work are applicable to other fields of medicine. Here we aim to provide practical information to aid clinicians in providing high-quality healthcare to Indigenous people. We start with a case vignette describing a situation that might be familiar to non-Indigenous clinicians caring for Indigenous patients.

## Case vignette

Robert is a general practitioner in a remote Australian town. He is seeing a 3-year-old girl, Sarah, with bronchiectasis who is attending clinic with her mother, Joanna. Robert feels that communication is challenging as Joanna does not seem to make eye contact and gives brief answers to questions. He also notices the child coughing whilst Joanna reports that she is not coughing. He feels as if he is unable to perform effectively as a doctor in relation to his patient. He knows he needs to gain Joanna's trust so he can effectively help Sarah. He shares his concerns with a colleague who recommends online training on culturally- safe provision of respiratory healthcare to Indigenous children.

A few weeks later Robert sees the patient with her mother in clinic again and adopts a culturally- safe approach. At this consultation he feels that engagement with Joanna is better, and he is obtaining a more accurate history. When talking through an information flip chart on lung health and explaining that chronic wet cough in a child can indicate chronic infection, Joanna asks “Why didn't you tell me this before? My child has had this wet cough for months!”.

Robert discusses treatment options with Joanna, and they agree on a 2-week regimen of antibiotics. Joanna asks several relevant questions about administration of the antibiotics and Robert feels more confident that she will administer the prescribed medications.

Key themes to improve engagement and outcome with Indigenous patients are presented below, followed by discussions of specific clinical scenarios.

## Indigenous families should receive culturally- safe healthcare

The provision of culturally- safe care leads to improved experiences and health outcomes (15). Culturally- safe care requires respect and understanding of Indigenous cultures and peoples (8). A culturally- safe environment helps Indigenous people to feel safe and respected, and facilitates their engagement with the healthcare provider, adherence to treatment, and clinical outcomes (8).

The ability to provide culturally- safe care requires, in the first instance, an understanding of the history of Indigenous peoples and knowledge of the ongoing effects of colonization. This includes an understanding of intergenerational trauma (15), often caused by government policies. Knowledge of Indigenous culture and the history of colonization promotes respect and understanding. Historical trauma and ongoing racism still have a profound effect on the interactions of Indigenous people with the healthcare system (16, 17). Recognition of ongoing systemic racism within society and within healthcare systems is essential for non-Indigenous people to understand why standard Western healthcare is often disempowering to Indigenous people (18). Furthermore, non-Indigenous clinicians must recognize the power imbalance between them and their patients (15). The following section expands on culturally-safe care.

## Culturally- safe health information

Providing health information in a way that is easily understood and relevant to Indigenous patients and their families is important for ensuring appropriate condition-specific health literacy. This is fundamental to enabling Indigenous patients to regain self-determination over medical care provided to them and/or their children. For non-Indigenous clinicians taking an accurate lung health history can be challenging. Patient/parental report of symptoms might not always reflect clinical findings (19). However, when a culturally- safe approach is combined with the use of health information resources, parental reporting of symptoms correlates well with clinical findings, e.g., a culturally-safe approach, combined with condition-specific flip charts, found that a history of wet cough correlated well with respiratory clinicians identifying a wet cough during their physical examination (20, 21). Disease specific applications (apps) (e.g., the “Lung Health for Kids” app (22) or the multi-lingual app developed for asthma) are also useful with helping to improve health literacy (23).

From a community perspective, using health information resources [flip charts (21), posters, YouTube videos (24), radio advertising (24), social media, and apps] that have been developed with Indigenous people are helpful with disseminating information related to specific health conditions. Use of such resources is associated with increased appropriate health seeking behavior (25), especially if they are developed with early input from end-users.

Condition-specific health literacy and knowledge also potentially improves adherence to prescribed treatment. A clinician cannot expect a parent to administer medicines to their child if they do not understand why the medicines are prescribed or whether the benefit outweighs the risk or opportunity cost (including the cost of not addressing more important priorities). Culturally- safe resources, such as flip charts, animated videos, and health apps are available for multiple respiratory and other conditions (20–22, 24, 26).

## Use of mobile phones to support clinical care

Effective communication between healthcare providers and patients is essential. The recent uptake of mobile phone use by isolated and disadvantaged families has dramatically (and unexpectedly) changed the options available. Clinical care provision can be enhanced with mobile phone communication, e.g., to liaise with patients/parents between appointments to facilitate treatment adherence (27). Using short message service (SMS) reminders for attending appointments as part of a wider strategy improves clinic attendance and clinical outcomes (28). It is important to remember that people might be reluctant to answer the phone when no caller identification is visible (29). Hence, having caller identification visible and texting before making phone calls can aid communication. Healthcare staff must also recognize that the turnover of mobile phones can be high in some communities. Therefore, collecting more than one mobile phone number (e.g., from close family members) is often useful for contacting patients. When phone communication fails, the local clinic and Indigenous staff can help facilitate communication with patients, as well as assisting with their care.

## Making the clinical environment (primary care clinics and hospitals) culturally-safe

As mentioned previously, the Western healthcare environment is often one in which Indigenous people feel uncomfortable (and may prefer to avoid). Understanding that Indigenous people may experience racism and discrimination (which influences their health-related quality-of-life) is vital (3, 30). Creating a culturally-safe healthcare environment requires leadership within the organization and close collaboration with local Indigenous people. Basic examples of how healthcare environments can be made to be more culturally-safe include the placement of Indigenous emblems and imagery (e.g., the Aboriginal and Torres Strait Islander flags or artwork), the creation of outdoor spaces, offering the use of interpreters (31), and ensuring that all staff have participated in cultural safety training. The presence of Indigenous health practitioners (see below) can greatly improve patient care.

When managing chronic health conditions, systems should allow the same clinician who undertook the initial consultation to follow their same patients (12). This allows the building of a relationship between the clinician and patient and facilitates the tracking of chronic symptoms.

## Improving in-hospital care to achieve better outcomes post-hospitalisation

Healthcare systems are complex. The hospital setting is further complicated by large clinical teams entering the patient's room (patients often find this intimidating), Indigenous people not being identified as Indigenous (hence not receiving culturally-safe care), and sub-optimal communication with the primary healthcare provider and clinic after discharge (11). We identified several steps (outlined in Table 1) that must occur before hospital discharge to ensure Indigenous children with chest infections attend subsequent clinic appointments and receive optimal ongoing management (11). By addressing these steps, we were able to demonstrate higher follow-up rates and improved respiratory health outcomes well after the hospitalisation episode (28).

**Table 1:** Steps to ensure optimal follow-up of Indigenous children hospitalised with a chest infection.

Step	Enablers
• Identifying and documenting the child is Indigenous	Admissions clerks should ask about Indigenous status even if patient does not appear Indigenous from their perspective
• Ensuring the family feels safe in hospital	Culturally-safe environment and approach essential
• Making sure the local clinic is named in the medical record when a primary healthcare provider is not identified	Ask about the name of the clinic when patient/parent cannot recall the name of the regular general practitioner
• Completing the discharge summary with follow-up instructions for the local healthcare provider or clinic	Clear follow-up instructions essential
• Taking steps to ensure the discharge summary reaches the local healthcare provider or clinic	Discharge summary has to be completed in time and sent to the correct address
• Providing the patients/caregivers with condition-specific health information in a culturally-safe manner, including the importance of attending follow-up appointments	Health information resources like flip charts, videos, apps, leaflets
• Sending short messaging service (SMS) reminders of appointments	If possible collect telephone numbers from patient and close family members.

From Laird et al. (11).

## Indigenous health practitioners facilitate clinical care

Patients living in rural or remote regions often need to undertake prolonged travel to reach specialist appointments in cities. This requires familiarity with travel (e.g., airport procedures). It is often complicated by financial hardship and complex socio-economic challenges that make clinic attendance an achievement to be acknowledged. Engaging with local supports, such as an Indigenous Health Practitioner or other resources within a service (e.g., the nurse who undertook the outreach service to the patient's community), can significantly reduce the stress experienced by the patient and their families. Indigenous Health Practitioners in Australia are primary health care professionals who work collaboratively within multidisciplinary healthcare teams to provide culturally-safe clinical care to Aboriginal and Torres Strait Islander people and communities. They are required to be registered with the Aboriginal and Torres Strait Islander Health Practice Board of Australia. This requires them to complete at minimum a 12-month Certificate IV program of study. They are uniquely placed to assist with cultural brokerage and allow context-specific adaptations to routine health service practices through Indigenous strengths-based ways of knowing, being and doing (32). Hence, they play a vital role in providing healthcare to Indigenous people. Their knowledge of the communities and family groups is important and will help non-Indigenous clinicians to understand the current health issues facing Indigenous people in these settings. Indigenous staff often facilitate referrals, clinic attendance, help clinics to function better, and enhance adherence to treatment. It is often valuable if the Indigenous Healthcare Practitioner can introduce the patient/parents to individual clinicians and explain their role.

## Common clinical scenarios

Certain aspects of practical advice for non-Indigenous clinicians providing care for Indigenous patients can be conveyed in the context of clinical scenarios. The clinical scenarios were identified by the clinicians on our CRE who collectively have many decades of experience in the field. The following are common scenarios, open to misinterpretation by non-Indigenous clinicians:

### Scenario 1: non-attendance at clinic

Many factors could potentially lead to non-attendance (33). Firstly, the patient/family might not have received the appointment details. Indigenous families often have responsibilities to travel to funerals and cultural events. Appointment letters do not always make their way to the intended recipient and thus sending a written letter for an appointment directly to the patient without notifying the local clinic may lead to a misunderstanding of “non-attendance”. Mobile phone communication (see above) and communication with the local clinic (for patients living in rural and remote areas) can facilitate communication and attendance of appointments (27). Communication might include giving the name of the doctor or team providing the clinic, the reason for the appointment, and providing some flexibility with scheduling the time of the appointment.

Patients might have to balance clinic attendance with other family related priorities. Patients might not be able to attend the clinic because of competing responsibilities at home. Mothers or other family members who bring children to the clinic often have other children in their care. Similarly, patients and/or parents frequently have responsibilities to other family members who need their help or support. The burden of chronic disease in Indigenous people is typically much higher than the general population (1). Illness of the parent or carer could also be a reason for non-attendance.

Indigenous people also have cultural responsibilities that they are expected to fulfill, e.g., attendance at funerals and men's business (a traditional event which generally occurs annually where boys are initiated into manhood in a cycle of ceremonies). Finally, transport issues and financial hardship are prevalent in Indigenous communities and can affect clinic attendance (33). This should be borne in mind when expecting patients to take time off work and travel to attend clinic.

Flexibility and good communication are required within the health system to assist clinic attendance. Patients and parents empowered with good understanding of health issues are more likely to seek healthcare (25), attend clinic (28) and make informed decisions about clinic attendance when weighing up competing demands.

Hence, the understanding of patients and their families of a health condition, and the importance of the clinic appointment are essential. The provision of health information and facilitation by Indigenous health practitioners (to explain why the appointment is necessary) are particularly important. Steps to facilitate clinic attendance are summarized in Box 1.

Box 1 **Steps to ensure optimal follow-up of Indigenous children hospitalised with a chest infection.**

• Patient given culturally-safe health information and understands the need for clinic attendance.
• Appointment letter (with details of the healthcare provider and/or team) sent to patient and to the local clinic.
• Mobile phone communication with patient and/or clinic (ensure caller identity is visible).
• Indigenous healthcare practitioners can facilitate clinic attendance.
• Flexibility with appointment scheduling.
• Provide help with travel to the clinic or consider local follow-up options.
• Be understanding if family or cultural obligations are barriers to clinic attendance.

From Laird et al. (11).

### Scenario 2: child attending healthcare appointment with a family member instead of biological mother

The Indigenous family structure is different from the traditional Western family structure and involves a kinship system (34). Aunts and grandmothers often take the role of the mother at various times in the child's life. Therefore, a child may attend appointments with an aunt or grandmother. Other reasons for the biological mother not attending are similar to the reasons cited above for non-attendance. Biological mothers might have other children whom they feel they cannot leave on their own while they bring a sibling to a healthcare appointment.

### Scenario 3: patient/family perceived not to be engaging with the clinician during the consultation

Recognizing the power imbalance between non-Indigenous health professionals trained in Western medicine over Indigenous patients can be challenging. In the above case vignette Dr. Robert struggled to engage with Joanna. One reason could be that Joanna, as an Indigenous woman, is (for cultural reasons) not allowed to make eye contact with a male she does not know. Other possible reasons for being perceived as not engaging include fear and distrust of the doctor (and/or the healthcare system) and the perception (that could be accurate) of a hostile clinical environment (35). Moreover, patients and their parents might feel overwhelmed by having to face the multiple challenges posed by society, navigation of a complex health system, and the burden of disease. This will often be compounded by cultural barriers and language. English may not be a first language for Indigenous people. Hence, one cannot assume an Indigenous patient/parent is proficient in English. This again highlights the need for culturally-safe communication and offering interpreters to overcome the communication divide (31). In addition to the above, clinicians should be aware of the high rates of hearing loss in Indigenous adults (36) and that hearing loss in a patient/parent could potentially impact a clinical consultation.

### Scenario 4: not filling prescriptions or adhering to the prescribed treatment regimen

Adherence to prescribed treatment can be challenging to measure accurately. Whilst there is no firm evidence that adherence to treatment is lower in Indigenous patients (37), a clinician might suspect that patients are not adhering to their treatment. Barriers to adherence are many. In addition to the barriers faced by the general population, Indigenous patients might also have sociocultural obligations which are considered to take priority over taking medicines, and barriers related to the cost of medicines, and the lack of appropriate storage facility (a working refrigerator for example). In a setting where there is fear and distrust, (see section above *Indigenous families should receive culturally-safe healthcare*) patients and parents must understand the condition being treated and the need for medication. Also important, patients might stop chronic medications once symptoms resolve. Here again culturally-safe communication and provision of health information are crucial (37).

In settings where there are competing demands and social challenges the involvement of family support and/or community members in medication dispensing is encouraged (38, 39). Increased availability of Indigenous health-care practitioners (40), the simplification of treatment regimens, and addressing socio-economic determinants of health can also facilitate adherence (37).

### Scenario 5: clinician not being able to contact the patient/family

A case is made above to use mobile phone communication to enhance the provision of clinical care. However, at times Indigenous patients or parents might not be contactable. Whilst challenges to making contact might be unique to individual people, some possible reasons for being difficult to contact are socio-cultural. Some Indigenous people have expressed reluctance to answer phone calls when caller is not identifiable (29). Patients might not be able to call back if they have run out of phone credit or if the phone is lost. Finally, patients and their families might be “on country” and have no phone reception. Spending time on country is thought to be a fundamental component of Aboriginal peoples’ wellbeing (41). Hence, understanding reasons why patients might be hard to contact helps prevent frustration when trying to facilitate care.

## Conclusion

Health disparities between Indigenous people and the non-Indigenous Australian population are immense, and so are the challenges faced by Indigenous people. A “business as usual” approach is insufficient when providing clinical care to Indigenous patients. With comprehensive, culturally-safe approaches to patient care measurable improvements can be made in the provision of healthcare and in health outcomes. To this end many training resources are available for clinicians (42, 43). Importantly, robust training should also be provided in medical, nursing, and allied health schools. A good starting point for non-Indigenous clinicians when clinical interaction with Indigenous patients and their families are not progressing as planned is to ask “What I am doing wrong and what can I be doing better?”.

## Data Availability

The original contributions presented in the study are included in the article/Supplementary Material, further inquiries can be directed to the corresponding author.

## Group members of The CRE Extended Group

CRE extended group are other investigators in the grant, higher degree research scholars, staff, collaborators and parents who attended the workshop held at the Children's Centre for Health Research, Brisbane on 24–26th August 2023. They contributed to the grant and/or the discussions that helped formulate this paper, read and contributed to the final manuscript: Atack JM, Azam S, Baxter C, Binks M, Bowden E, Bui DS, Cheng AC, Dharmage S, Gadoury A, Garriga-Grimau L, Grimwood K, Hill J, Hoffman LR, Howard DR, Fuery A, Kapur N, Kok HC, Laird P, Lau G, Mandal PK, Marsh R, McElrea MS, McPhail SM, Mitchell R, Morris P, Petsky HL, Perret JL, Pratt A, Ramsey KA, Roberts JM, Robinson PD, Rodwell LT, Torzillo P, Vicendese D, West NP, Wurzel D, Yerkovich S.
